# Hepatitis E prevalence in a sexual high-risk population compared to the general population

**DOI:** 10.1371/journal.pone.0191798

**Published:** 2018-01-25

**Authors:** Jeanne Heil, Christian J. P. A. Hoebe, Inge H. M. van Loo, Jochen W. L. Cals, Geneviève A. F. S. van Liere, Nicole H. T. M. Dukers-Muijrers

**Affiliations:** 1 Department of Sexual Health, Infectious Diseases and Environmental Health, Public Health Service (GGD) South Limburg, Geleen, the Netherlands; 2 Department of Medical Microbiology, Care and Public Health Research Institute (CAPHRI), Maastricht University Medical Center (MUMC+), Maastricht, the Netherlands; 3 Department of Family Medicine, Care and Public Health Research Institute (CAPHRI), Maastricht University, Maastricht, the Netherlands; Centers for Disease Control and Prevention, UNITED STATES

## Abstract

Transmission routes of the hepatitis E virus (HEV) are under debate. Here, we studied possible sexual transmission by comparing HEV prevalence in a Dutch sexual high-risk population (n = 1,482) with that in a general population (n = 1,487) while assessing sociodemographic and sexual risk factors. Overall prevalence of anti-HEV IgG of 18.9% (n = 562) was, adjusting for confounders, similar between the two populations (p = 0.44). Prevalence was higher with each year’s increase in age (adjusted OR: 1.03, 95%CI: 1.02–1.04, p<0.01), among men (adjusted OR: 1.24, 95%CI: 1.02–1.50, p = 0.03) and among individuals diagnosed with sexually transmitted infections (adjusted OR: 1.60, 95%CI: 1.02–2.49, p = 0.04). Our results only hint at the possibility of a sexual transmission route for HEV given higher rates in those with chlamydia and/or gonorrheal infections. Sexual transmission is not a dominant transmission route, as its prevalence was not higher for the sexual high-risk population than for the general population.

## Introduction

Recently, the hepatitis E virus (HEV) has become a global public health concern, as an increase of autochthonous HEV infections was observed in developed countries. HEV is considered to be endemic in developing countries, where it is transmitted mainly through contaminated water and the faecal-oral route, causing epidemic outbreaks of genotypes 1 and 2 [1, 2]. Traditionally, HEV was considered to be a travel-related disease in developed countries, associated with genotypes 1 and 2. For genotypes 1 and 2, the mortality rate is usually low (0.07–0.6%); it is particularly severe among pregnant women, with mortality rates between 15–25% [3]. However, reported cases of patients that have not travelled to endemic areas—so-called autochthonous hepatitis infections—have increased in developed countries [4–10]. These infections are mainly caused by HEV genotype 3, symptomatic HEV genotype 3 infections are more common among middle-aged and older individuals as well as among men [10–12]. HEV infections are usually asymptomatic, but genotype 3 infections may cause chronic hepatitis in immunocompromised patients. This group includes patients who have received an organ transplant, patients receiving chemotherapy and HIV-infected individuals [13–16].

Transmission routes continue to be one of the most debated aspects of HEV. The most recent evidence suggest that HEV caused by genotypes 3 and 4 is transmitted zoonotically. In Europe, North America and Japan, HEV genotype 3 is widespread among pigs [17–19]. In these countries, domestic pigs and wild boars act as a reservoir [20]. It is proven that HEV is transmitted by ingesting uncooked or poorly cooked pork or game meat [21–23].

Studies are needed to identify all transmission routes of HEV, so appropriate preventive and control measures can be taken. The hepatitis A virus (HAV) and HEV genotypes 1 and 2 are both single-stranded RNA viruses with similar incubation periods and a faecal-oral transmission route. Sexual activity, including oral-anal contact, as the major transmission route among men who have sex with men (MSM) is another widely documented aspect of HAV [24, 25], but only a limited number of studies have focused on the possible sexual transmission route of HEV infections [26–30]. This study could contribute to policy on the prevention and control of HEV infections, to be targeted at vulnerable individuals who are at highest risk.

To explore the possible role of sexual transmission, we compared the HEV prevalence of a population with higher sexual risk to the general population. In addition, we assessed potential risk factors of sexual transmission through a cross-sectional study in the south of the Netherlands.

## Methods

### Study population

The STI clinic study and the GP study were approved by the Maastricht University Medical Centre Medical Ethical Committee (11-4-108 for the STI clinic cohort and 14-4-042 for the GP cohort). No informed consent was needed because of prevailing laws in the Netherlands, as it concerns an observational study using anonymous data only. Our study population consisted of two populations: a sexual high-risk population from a STI clinic cohort and the general population from a GP cohort. Venous blood samples were tested on HEV IgG. All individuals from the two study populations lived in urban areas in South Limburg. In the study period (December 2011 to November 2015), no clusters or outbreaks of HEV were reported in the study region.

First, the sexual high-risk study population (STI clinic cohort; n = 1,482) was compiled as a sample from a cohort of individuals aged 20–70, all of whom were tested for a sexually transmitted infection (STI) at the STI clinic between December 2011 and November 2015 (original cohort size about 24,500 individuals). This sample included women (n = 350); female swingers, heterosexuals who as a couple practise partner-swapping or group sex, or who visit sex clubs for couples [31] (n = 184); heterosexual men (n = 480); and men who have sex with men (MSM; n = 468). This STI clinic cohort represented a high-risk population, as 75.2% reported anal sex, had three or more sexual partners in the past six months or tested positive for *Chlamydia trachomatis* (CT), *Neisseria gonorrhoeae* (NG), syphilis or HIV (see S1 Table).

Second, the general study population (GP cohort; n = 1,487) was compiled as a sample from a cohort of individuals aged 40–70, all of whom were tested by general practitioners (GPs) for a study of the hepatitis B virus (HBV) and the hepatitis C virus (HCV) between September 2014 and April 2015 (original study size n = 3,434). The prevalence of past HCV infections was 0.27% (4/1,487). This is comparable to the prevalence in the general population [32–34]. No active HCV infections were diagnosed in the GP cohort. The prevalence of active and past HBV infections was 0.5% (7/1,487) and 8.9% (132/1,487) respectively. Overall, the prevalence of active and past HBV infections is 0.2–0.3% [35, 36] and 2.1% [36] in the Netherlands. This GP cohort represented a general population of both man and women from two urban areas, without any further exclusion criteria.

### Data collection

Data on the test year, age, gender, ethnicity, HIV positivity and anti-HEV test results were available for all samples. In the STI clinic cohort, HIV and syphilis positivity was based on the testing of high-risk groups such as MSM. In the GP cohort, HIV positivity was based on self-reported data.

Additional data on sexual preference, reported anal sex, number of sexual partners in the past six months and diagnosis of CT/NG/syphilis were only available for the STI clinic cohort. Additional data on educational level, HBV and HCV positivity were only available for the GP cohort. Reporting anal sex, having three or more sexual partners in the past six months and being CT positive, NG positive, Syphilis positive or HIV positive were categorised as risk factors of sexual transmission.

### Serological testing

Stored sera from the participants of the two study populations were tested for HEV IgG (*recom*Well hepatitis E virus IgG, Mikrogen GmbH, Neuried, Germany) at the Department of Medical Microbiology at Maastricht University Medical Center. HEV IgG was measured in U/ml: results below 20 U/ml were reported as negative, 20–24 U/ml (n = 41) were reported as grey areas (considered as negative in current analyses) and results above 24 U/ml were reported as positive. In a recent study, the Wantai assay detected more positives (prevalence of 48.2% compared to 17.3% with the Mikrogen assay) and had a lower detection limit. However, it is likely that this included more false positives and the Mikrogen assay had the highest correlation to the estimated overall IgG (prevalence of 18.7%). Therefore, we chose for the Mikrogen assay.

### Statistical analysis

**Fig 1:**
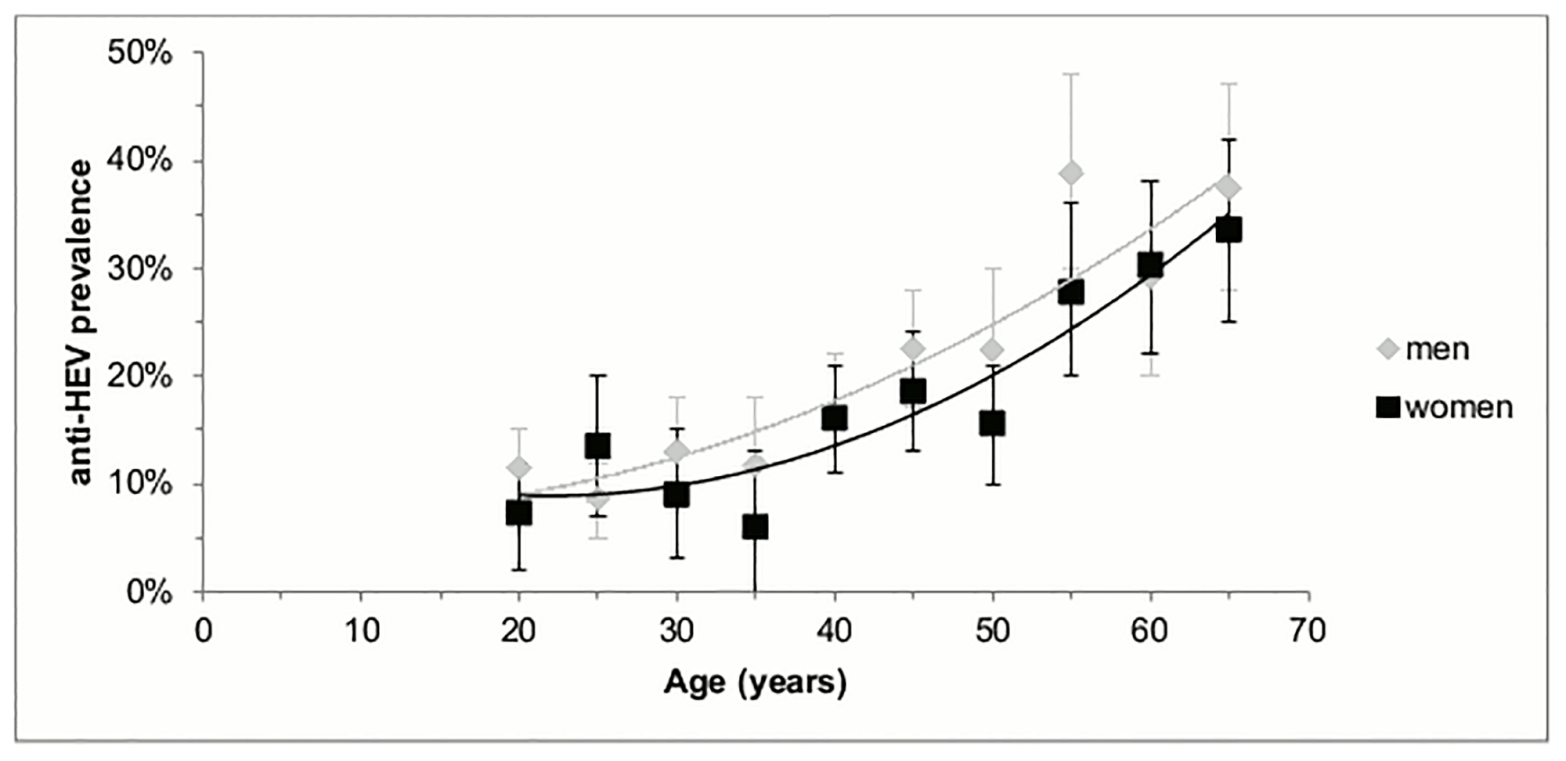
Anti-HEV IgG prevalence in five-year age groups (n = 2,969), south Netherlands, Dec 2011 to Nov 2015.

The prevalence of HEV was based on the number of positive HEV test results, divided by the total number of HEV tests and multiplied by 100. Three different models were constructed, including an overall model, a model for the STI clinic cohort and a model for the GP cohort. Univariate logistic regression analyses were used to assess the unadjusted associations between potential risk factors and HEV prevalence. All previously described variables (see the Data collection section) were included in the analyses as potential risk factors. However, we identified several known confounders on the basis of the literature, which led to adjustments in the multivariate logistic regression analyses. These confounders include the test year, age and gender. We used the Enter method to assess the OR and the 95% confidence intervals (p≤0.05). The age curves in Fig 1 were designed on the basis of a second-degree polynomial fit.

## Results

A total of 2,969 individuals were tested on HEV IgG antibodies: 1,482 from the STI clinic cohort and 1,487 from the GP cohort. The study population had a median age of 44 (IQR 30–54), 54.9% were men (n = 1,630) and 11.0% (n = 326) had a non-western ethnicity (see S1 Table). Anti-HEV IgG was detected in 562 samples (18.9%, 95%CI: 17.5–20.4).

### Univariate risk factors

In univariate analyses, the overall HEV prevalence was higher with each year’s increase in age (p<0.01) as well as for individuals from the GP cohort (p<0.01; see Table 1). For the STI clinic cohort, HEV prevalence was higher with each year’s increase in age (p<0.01), for MSM (p = 0.04), for individuals who reported anal sex (p = 0.02), for individuals who had three or more sexual partners (p = 0.05) and with a diagnosis of CT or NG (p = 0.05). For the GP cohort, the HEV prevalence was higher with each year’s increase in age (p<0.01) and borderline significant for men (p = 0.10).

**Table 1:** Determinants of anti-HEV IgG prevalence, south Netherlands, Dec 2011 to Nov 2015.

Total study population (n = 2,969)	Univariate anti-HEV IgG prevalence		Multivariate OR (95%CI) adjusted for test year^a^, age, gender
Age (median = 44, IQR = 30–54)		***	1.03*** (1.02–1.04)
Gender			
- Men	309/1,630	19.0%	1.24* (1.02–1.50)
- Women	253/1,339	18.9% (ref)	1.00
Population			
- High sexual risk (STI clinic cohort)	176/1,482	11.9% (ref)	1.00
- General (GP cohort)	386/1,487	26.0%***	1.19 (0.76–1.87)
*STI clinic cohort* (*n = 1*,*482*)			adjusted for test year^a^, age, sexual preference
Age (median = 30, IQR = 24–39)		***	1.02** (1.01–1.04)
Gender			
- Men	118/948	12.4%	1.23 (0.88–1.73)
- Women	58/534	10.9% (ref)	1.00
Sexual preference			
- Women	33/350	9.4% (ref)	1.00
- Female swinger	25/184	13.6%	1.17 (0.65–2.11)
- Heterosexual men	52/480	10.8%	1.19 (0.75–1.89)
- MSM	66/468	14.1%*	1.41 (0.90–2.23)
Reported anal sex			
- Yes	87/629	13.8%*	1.27 (0.86–1.88)
- No	81/812	10.0% (ref)	1.00
- Unknown	8/41	19.5%^#^	2.11^#^ (0.92–4.83)
Number of sexual partners (median = 3, IQR = 2–5)			
- <3 in past 6 months	61/617	9.9% (ref)	1.00
- ≥3 in past 6 months	114/856	13.3%*	1.31 (0.93–1.85)
- Unknown	1/9	11.1%	1.09 (0.13–9.03)
Chlamydia and/or gonorrhoeae positive			
- Yes	28/171	16.4%*	1.60* (1.02–2.49)
- No^b^	148/1,311	11.3% (ref)	1.00
Syphilis positive			
- Yes	1/4	25.0%	1.75 (0.16–18.72)
- No	14/114	12.3% (ref)	1.00
- Untested	161/1,364	11.8%	0.88 (0.46–1.68)
HIV positive			
- Yes	6/37	16.2%	1.08 (0.43–2.74)
- No	167/1,427	11.7% (ref)	1.00
- Untested	3/18	16.7%	1.48 (0.42–5.22)
*GP cohort* (*n = 1*,*487*)			adjusted for test year^a^, age, gender
Age (median = 53, IQR = 46–62)		***	1.04*** (1.03–1.05)
Gender			
- Men	191/682	28.0%^#^	1.25^#^ (0.99–1.58)
- Women	195/805	24.2% (ref)	1.00
Educational level			
- Low	185/676	27.4%	1.12 (0.82–1.51)
- Medium	94/371	25.3%	1.11 (0.80–1.55)
- High	102/427	23.9%(ref)	1.00
- Unknown	5/13	38.5%	1.43 (0.45–4.55)
HIV positive			
- Yes	1/5	20.0% (ref)	1.00
- No	352/1,374	25.6%	1.46 (0.16–13.42)
- Untested	33/108	30.6%	1.91 (0.20–18.21)
HBV positive			
- Yes (active or past infection)	43/139	30.9%	1.15 (0.78–1.70)
- No (no infection or vaccinated)	343/1348	25.4% (ref)	1.00
HCV positive			
- Yes (past infection)	3/4	75.0%^#^	8.77^#^ (0.89–86.40)
- No (no infection)	383/1483	25.8% (ref)	1.00

^a^ Test year of 2012 includes two tests from December 2011.

^b^ Including 14 individuals not tested for CT and 13 individuals not tested for NG.

*p≤0.05

**p≤0.01

***p≤0.001

^#^0.05≤p≤0.10

### Independent risk factors

In multivariate analyses, the overall HEV prevalence was higher with each year’s increase in age (adjusted OR: 1.03, 95%CI: 1.02–1.04, p<0.01) and for men as compared to women (adjusted OR: 1.24, 95%CI: 1.02–1.50, p = 0.03; see Table 1 and Fig 1). HEV prevalence was similar among the STI clinic cohort and the GP cohort, after adjusting for confounding factors (see Table 1).

For the STI clinic cohort, most univariate sexual risk factors attenuated and became statistically non-significant after adjusting for test year, age and sexual preference. Independent predictors for HEV prevalence were older age (adjusted OR: 1.02, 95%CI: 1.01–1.04, p<0.01) and a diagnosis of CT or NG (adjusted OR: 1.60, 95%CI: 1.02–2.49, p = 0.04).

For the GP cohort, HEV prevalence was higher with each year’s increase in age (adjusted OR: 1.04, 95%CI: 1.03–1.05, p<0.01).

## Discussion

No firm conclusions can be drawn about sexual transmission of HEV from this study given that the overall prevalence of anti-HEV IgG was the same between the sexual high-risk population and the general population. Our results only hint at the possibility of sexual transmission of HEV as having a sexually transmitted infection (CT and/or NG) was found to be associated with a higher HEV prevalence. Though this may lead to occasional HEV outbreaks, as is the case for HAV, sexual HEV transmission is unlikely to influence the prevalence at the population level. More research is needed on possible sexual transmission of HEV.

Anti-HEV IgG was detected in 18.9% of the 2,969 blood samples from individuals either attending an STI clinic or belonging to the general population. The adjusted HEV prevalence was similar among the two cohorts. This observed prevalence is similar to the figure of 17% found in a German study among persons aged 18–79 years, which used the same HEV assay [37]. The observed prevalence is lower than a study among Dutch blood donors with a prevalence of 27% [11]. This difference in HEV prevalence is unlikely only explained by the use of the Wantai assay with a slightly higher sensitivity [38], it is more likely explained by the older study population. The identification of age and gender factors with relation to HEV prevalence confirms the results from several previous studies [10–12, 39]. HEV prevalence is thought to be related to age, because of the cumulative effect of lifetime exposure. However, no clear and thorough explanation has as yet been found for its connection with gender.

Interestingly, HEV prevalence was higher for individuals who tested positive for CT and/or NG. This higher prevalence is unlikely explained by the zoonotic transmission route of HEV. The ingestion of HEV-infected food is not believed to be more common among individuals who tested positive for CT and/or NG. Furthermore, a similar trend is seen in other risk factors of sexual transmission such as being an MSM, reporting anal sex, having three or more sexual partners in the past six months and being tested positive for syphilis. This may suggest that sex may be a possible transmission route for HEV. Whether HEV could be transmitted via the faecal-oral route, via blood contact during risky sexual behaviour or via person-to-person contact remains unknown and should be the subject of further study.

To our knowledge, this is the first study to explore the possible sexual transmission of HEV and its impact at the population level in the Netherlands. However, the following limitations must be considered. First, no data on sexual risk behaviour were available for individuals from the GP cohort and data on parenteral risk behaviour, injective drug use, were not included due to its low prevalence. However, we compared the sexual risk behaviour of the STI clinic cohort to the general population in the Netherlands. In our study 40% of the individuals reported anal sex in the past six months compared to 7–18% in the general population [40, 41]. Overall more sexual risk behaviour was reported in the STI clinic cohort compared to the general population. Second, the age distribution differs between the STI clinic cohort and the GP cohort. Age was included as confounder in the multivariate logistic regression analyses. Additionally, we assessed the HEV prevalence in the 40–50 year olds. No difference was detected between the STI clinic cohort and the GP cohort in this age group too, p = 0.678. Matching cases and controls on age and/or sex is desirable for future studies[26, 27]. Third, the comparison to HEV prevalence in other studies is difficult to make, as sensitivity and specificity are highly variable between the different serological assays [42, 43]. Fourth, no data on occupational exposure to swine, as a known risk factor of HEV, were available. Finally, using this cross-sectional design we were unable to assess causal associations, a longitudinal study including genotyping would have been the ideal design. Our results only hint at the possibility of a sexual transmission route for HEV given higher rates in those with chlamydia and/or gonorrheal infections. Sexual transmission is not a dominant transmission route, as its prevalence was not higher for the STI clinic cohort than for the GP cohort. Whether sexual transmission of HEV occurs, and whether it could consequently lead to outbreaks in the same vein as HAV, should preferably be studied at the individual level in future prospective research. Considering the limited clinical impact of HEV infections, we do not recommend increased HEV testing in sexual high-risk clinical settings, with the possible exception of patients who are immunocompromised.

## Supporting information

S1 Table Characteristics of total samples tested for HEV (n = 2,969), south Netherlands, Dec 2011 to Nov 2015^a^. (DOCX)
