# Supplementary material for: Hepatitis E prevalence in a sexual high-risk population compared to the general population
Source: PLoS One. 2018 Jan 25;13(1):e0191798. doi: 10.1371/journal.pone.0191798 (PMC5784977; doi:10.1371/journal.pone.0191798)
Supplement: S1 Table — (DOCX) [file pone.0191798.s001.docx]

**S1 Table. Characteristics of total samples tested for HEV (n=2,969), south Netherlands, Dec 2011 to Nov 2015^a^**

|  | Sexual high-risk population  N=1,482  % (n) | General population  N=1,487  % (n) | Total  N=2,969  % (n) |
| --- | --- | --- | --- |
| Age | median=30, IQR=24-39 | median=53, IQR=46-62 | median=44, IQR=30-54 |
| Gender |  |  |  |
| - Men | 64.0 (948) | 45.9 (682) | 54.9 (1,630) |
| - Women | 36.0 (534) | 54.1 (805) | 45.1 (1,339) |
| Non-western ethnicity^b^ |  |  |  |
| - Yes | 13.4 (199) | 8.5 (127) | 11.0 (326) |
| - No | 86.6 (1,283) | 91.5 (1360) | 89.0 (2,643) |
| *Additional variables only available for STI clinic cohort* | | | |
| Sexual preference |  |  |  |
| - Women | 23.6 (350) | - |  |
| - Female swinger | 12.4 (184) |  |  |
| - Heterosexual men | 32.4 (480) |  |  |
| - MSM | 31.6 (468) |  |  |
| Sexual transmission risk factor^c^ |  |  |  |
| - Yes | 75.2 (1,114) | - |  |
| - No^d^ | 24.8 (368) |  |  |
| *Additional variables only available for GP cohort* | | | |
| Educational level |  |  |  |
| - Low | - | 45.5 (676) |  |
| - Medium |  | 24.9 (371) |  |
| - High |  | 28.7 (427) |  |
| - Unknown |  | 0.9 (13) |  |

^a^ Test year of 2012 includes two tests from December 2011.

^b^ individuals who were born in or who had at least one parent born in Africa, Asia (excluding Indonesia and Japan) or Latin-America.

^c^ Including reporting anal sex, having three or more sexual partners in the past six months and being CT positive, NG positive, Syphilis positive or HIV positive.

^d^ Including five tests for which the risk factor was unknown.
